# Toward a gut-AMPK axis: the microbiome and AMPK signaling in nutrition and healthy aging

**DOI:** 10.3389/fphys.2026.1861013

**Published:** 2026-07-03

**Authors:** Erik Dassoff, Hashim Islam, Jacob Allen

**Affiliations:** 1 Independent Researcher, Tacoma, WA, United States; 2 School of Health and Exercise Science, The University of British Columbia Okanagan, Kelowna, BC, Canada; 3 Center for Chronic Disease Prevention and Management, The University of British Columbia Okanagan, Kelowna, BC, Canada; 4 Department of Health and Kinesiology, University of Illinois at Urbana-Champaign, Urbana, IL, United States

**Keywords:** aging, AMPK, bioenergetics, gut - brain axis, healthspan, metabolism, microbiome, nutrition

## Abstract

Modern nutrition research has evolved from early discoveries of essential vitamins to exploring the complex interactions between a much larger range of food-derived compounds (the foodome). The microbiome is a key link between diet and health outcomes through multiple gut-host axes. However, the field lacks a unifying framework to demystify its inherent complexities. This review outlines mechanistic evidence that the 5’-AMP-activated protein kinase (AMPK) is a potential mediator of host-microbe relationships and helps move towards the concept of a gut-AMPK axis. Further, it suggests that a plausible function of a “healthy” gut microbiome is to increase the bioavailability of metabolites capable of modulating AMPK. These insights can facilitate larger efforts to define what constitutes a functional gut microbiome, understand the health effects of the broader foodome, and support organismal resilience and healthy aging.

## Graphical Abstract

**Figure:**
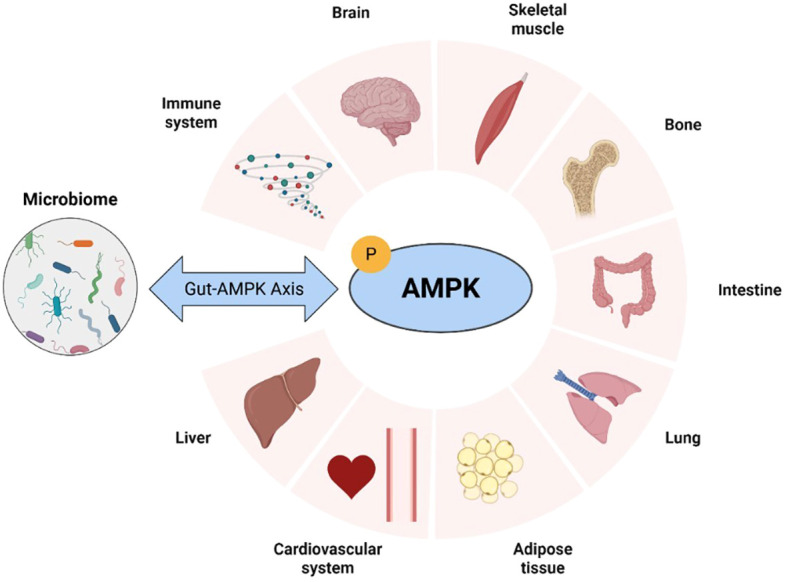

## 1 Introduction

Nutrition research might be considered to have begun as early as the mid-1800s, with the discovery that lemons could cure scurvy (Mozaffarian et al., 2018). We have since established the importance of a wide range of vitamins and minerals and made great strides in understanding how dietary patterns impact health. Nonetheless, if the old adage, “you are what you eat,” still rings true, it underscores the need for deeper exploration.

Currently, nutrition science focuses heavily on approximately 150 nutritional components, with a select few being featured on the nutrition facts panel (Menichetti et al., 2025). However, more than 139,000 molecules have been documented in food ingredients (Menichetti et al., 2025). This leads to an emerging area of nutrition science focused on understanding the much broader range of compounds in our foods (termed the foodome) and their interactions with food processing, individual genetics, and the gut microbiome (Mozaffarian et al., 2018; Menichetti et al., 2025). The gut microbiome is a valuable starting point for the next generation of nutrition research, as it is reported to dictate about half of the quantifiable metabolites in our blood (Diener et al., 2022). Interpreting the effects of these metabolites could fill gaps in our understanding of how foods influence health and disease.

The gut microbiome has been linked to the health of numerous tissues through the gut-brain, gut-muscle, gut-liver, gut-lung, and gut-adipose axes, among others (Schroeder and Bäckhed, 2016). However, the microbiome’s complexity is both a benefit and a drawback. Thousands of metabolites offer the potential to expand our knowledge of nutrition. Still, accurately defining a “healthy” gut microbiome is a complex and challenging pursuit (Diacova et al., 2025).

Identifying a common thread linking multiple gut-host axes could streamline the research agenda and provide unprecedented insights into how the microbiome influences healthy aging. The 5’-adenosine monophosphate-activated protein kinase (AMPK) enzyme is a promising link. It is a highly conserved cellular energy sensor, universally present in eukaryotic cells (Hardie et al., 2016). Notably, AMPK activation has been linked to a broad range of health outcomes, including improved exercise performance (Niederberger et al., 2015) and sleep (Chikahisa et al., 2009), reduced depression (Fang et al., 2020) and anxiety (Zhang et al., 2023), and a reduced risk of neurodegenerative diseases, skeletal muscle atrophy, cancer, and cardiometabolic diseases (Canbolat and Cakıroglu, 2023; Steinberg and Hardie, 2023). AMPK’s potential to mitigate disease risk is likely due to its broad targeting of numerous anti-aging pathways, including anti-senescence, anti-oxidative, and anti-inflammatory effects, alongside reduced mTORC1 phosphorylation and enhanced DNA repair, phagocytosis, and efferocytosis (Bae et al., 2011; Salminen and Kaarniranta, 2012; Xu et al., 2023; Dassoff, 2026). However, an important caveat is that AMPK activation is not universally beneficial, and its effects can depend on factors such as timing, amplitude, energetic status, or other characteristics (Dassoff, 2026).

In this review, we aim to synthesize evidence suggesting that multiple microbially derived compounds may interact with AMPK and that such interactions may be relevant for nutrition and healthy aging. We will first introduce examples of microbially produced compounds with important links to human health. We then discuss their ability to modulate AMPK (primarily in animal models), discuss evidence for AMPK-dependent effects, and examine the proposed mechanisms of AMPK activation. Finally, we present a brief framework to guide future research—from discovering new compounds, to rapidly screening those with health-promoting potential, and ultimately validating them through clinical studies.

## 2 Methodology

This review was intended as an exploratory narrative review without a predefined search strategy. However, examples of searches are described for transparency. The search was conducted in Omni, an academic search tool provided by The Ontario Council of University Libraries. Search terms included combinations of key gut-derived metabolites (e.g., “indoles,” “short-chain fatty acids,” “equol,” etc.), “prebiotics,” “probiotics,” “microbiome,” and “AMPK” without an explicit emphasis on either human or animal studies. An effort was made to include trials that highlighted gut metabolite-AMPK relationships across multiple metabolite classes and tissues, including both peripheral and central effects. Conflicting information was included when uncovered (e.g., dihydro-resveratrol). Searches for translational trials incorporated terms pairing the metabolites of interest with systematic reviews of randomized controlled trials. Additional searches were conducted to locate articles describing supporting information.

## 3 Effects of the gut microbiome on AMPK modulation

Multiple lines of evidence suggest that microbially derived metabolites may interact with AMPK, with one striking example coming from fecal microbiota transplantation studies in mice. Microbiota from exercise-trained donors were transferred into sedentary germ-free recipients, after which the recipients exhibited increased skeletal muscle AMPKα expression (Kerr et al., 2023). Accordingly, the exercise-conditioned fecal microbiota subsequently improved glucose tolerance in mice fed a high-fat diet for 8 weeks (Kerr et al., 2023). This example supports the rationale for a gut–AMPK axis. It can be further expanded by examining how diverse microbiota-targeted interventions influence host AMPK activity and/or downstream processes across tissues. Notably, phosphorylation of AMPK at its threonine 172 (Thr172) residue relative to total AMPK is commonly used as a proxy for AMPK activity. However, it is not a direct equivalent and should be interpreted with caution.

### 3.1 Pre and probiotics

**Figure 1:**
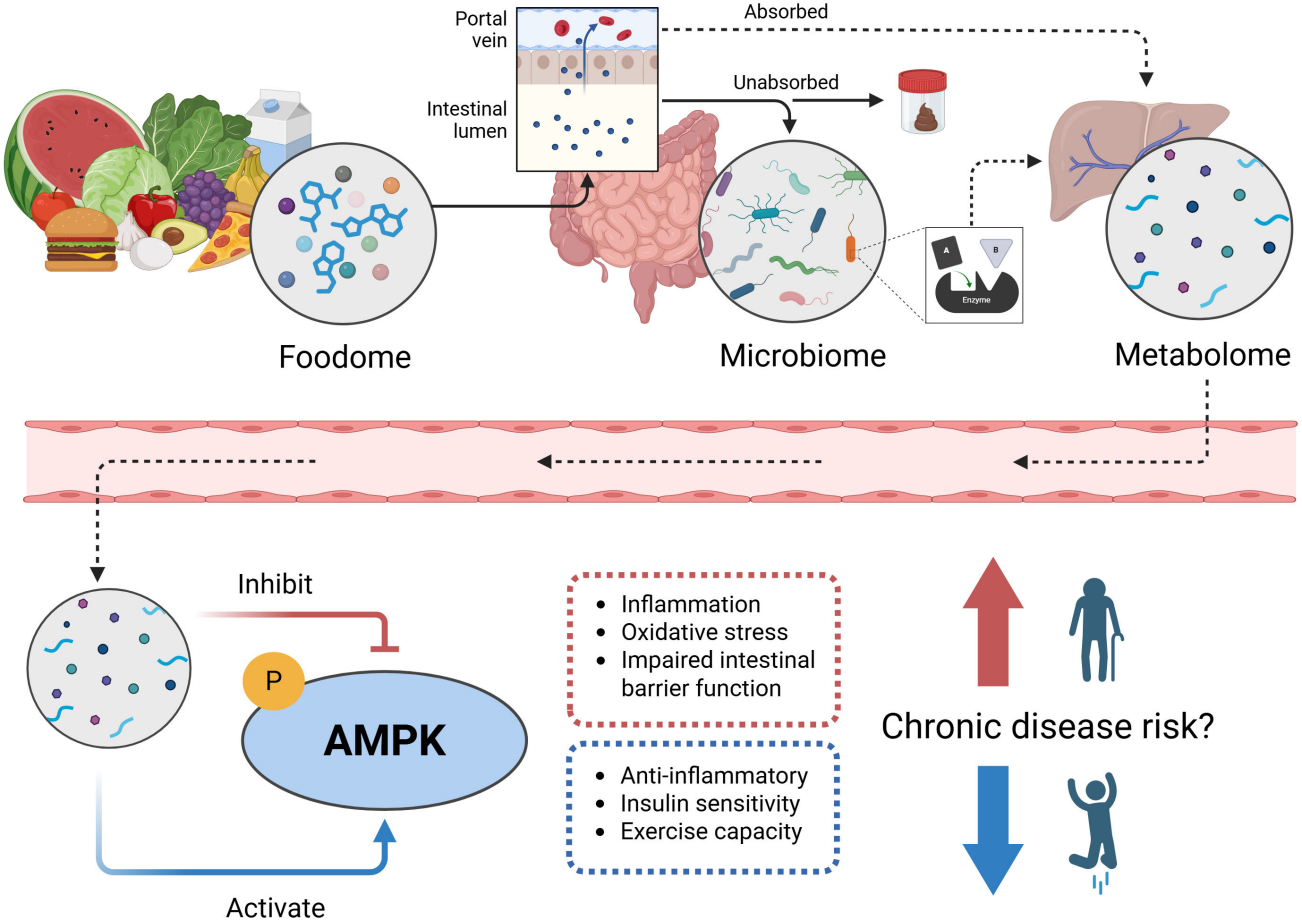
From foodome to metabolome and the impact of AMPK on chronic disease. There are approximately 139,000 documented compounds in foods (the foodome). These compounds are either absorbed into the portal vein and metabolized by the liver or travel to the microbiome for further enzymatic conversions by microorganisms. Gut metabolites can then be absorbed and metabolized by the liver or excreted in feces. Absorbed compounds make up the metabolome and can increase or decrease AMPK phosphorylation or expression or alter its sensitivity in response to other stimuli. Interactions of compounds with specific tissues may depend on barrier transport (i.e., the blood-brain barrier). AMPK activation may help modulate physiological processes that contribute to reduced chronic disease risk. Created in Biorender.com.

The gut microbiome may be thought of as an endogenous bioreactor that produces bioavailable and bioactive metabolites from foods that impact various aspects of health (**Figure 1**). The production of beneficial compounds could, therefore, be modulated by providing new microbial functions in the form of probiotics or by altering the substrates for microbes to feed on.

Whole-food products that naturally contain probiotics may offer one practical route for modulating host AMPK activity. In mice, consumption of a probiotic-rich cheese containing *Lactococcus lactis* LB1022 and *Lactiplantibacillus plantarum* LB1418 reduced the severity of alcohol-induced liver injury and increased hepatic AMPK expression (Kim et al., 2023). Similarly, feeding mice a probiotic yogurt (containing *Lactobacillus rhamnosus* HF01) increased liver AMPK activity and downstream expression of the acetyl-CoA carboxylase 1 (ACC1) gene in high-fat diet-fed obese mice (Sun et al., 2024).

Prebiotic substrates can similarly modulate AMPK by shaping microbial metabolism. Supplementing a high red meat diet in mice with soluble dietary fiber increased intestinal epithelial AMPK ß1/2 phosphorylation, alongside increased short-chain fatty acid production and decreased trimethylamine-N-oxide (TMAO) production (Li et al., 2017). However, studies assessing the effects of prebiotics and probiotics on AMPK are relatively scarce, whereas more data are available for the effects of specific microbial metabolites.

### 3.2 Postbiotics

Several major classes of microbial metabolites, including SCFAs, phytochemical derivatives, and secondary bile acids, have now been shown to engage AMPK-related pathways in distinct tissues (**Table 1**).

**Table 1:** Representative studies linking gut microbial derivatives to AMPK modulation from rodent and pre-clinical models.

Intervention	Model type (tissue)	Effect on AMPK	Associated phenotypic outcome	References
Polyphenol and other phytochemical derivatives				
Oral sulforaphane	High-fat diet-fed mice (liver)	↑ (p-AMPKα/t-AMPK)	↑ insulin sensitivity	Zhang et al. (2022)
Urolithin A-treated cells	Cultured C2C12 muscle cells	↑ (p-AMPK/t-AMPK)	↑ p62 and LC3-II flux	Moradi et al. (2025)
Oral urolithin A	Mice (cerebral cortex and hippocampus)	↑ (p-AMPK/t-AMPK)	↑ memory, ↓ neuron death	Gong et al. (2019)
Oral dihydro-resveratrol and treated cells	Obese mice (liver), cultured adipocytes and hepatocytes	↑ (p-AMPKα/t-AMPK)	↑ insulin sensitivity	Lam et al. (2023)
Intraperitoneal dihydro-resveratrol	High-fat, high-sugar-fed mice (liver)	↔ (p-AMPKα/t-AMPK)	↔ insulin sensitivity or plasma lipid profile	Pallauf et al
Oral equol and treated cells	Genetically-induced obese mice (skeletal muscle) and cultured myotubes	↑ (p-AMPKα/t-AMPK)	↑ glucose uptake and improved serum metabolic profile	Cheong et al. (2014)
Oral protocatechuic acid	Mice (skeletal muscle) and cultured myotubes	↑ (p-AMPKα/t-AMPK)	↑ oxidative capacity, mitochondrial biogenesis, and antioxidant capacity; type II to type I muscle fiber type conversion	Yang et al. (2023)
Oral p-coumaric acid and treated cells	Mice (liver and hypothalamus), cultured hypothalamic cells and hepatocytes	↑ (liver), ↓ (hypothalamus) (p-AMPKα/t-AMPK)	↑ insulin sensitivity and hypothalamic leptin sensitivity, ↓ food intake	Nguyen et al. (2021)
Short-chain fatty acids				
Intraperitoneal acetate	Mice (hypothalamus)	↓ (p-AMPKα/t-AMPK)	↑ satiety hormones, ↓ acute food intake	Frost et al. (2014)
Oral acetate and treated cells	ApoE-deficient mice and cultured macrophages	↑ (p-AMPKα/t-AMPK)	↓ atherosclerotic plaque formation	Wada et al. (2025)
Oral acetate and treated cells	Influenza A-infected mice (lung) and cultured bronchial epithelial cells	↑ (p-AMPKα/t-AMPK)	Improved airway epithelial barrier function	Hu et al. (2024)
Oral sodium butyrate	OVX-mice with diet-induced obesity (skeletal muscle)	↑ (p-AMPKα/t-AMPK)	↑ energy expenditure and insulin sensitivity, ↓ body fat	Fu et al. (2023)
Oral SCFA cocktail	Pre-sarcopenic senescence-accelerated mice (skeletal muscle)	↑ (p-AMPKα/t-AMPK)	↑ muscle mass, grip strength, anti-fatigue capacity	Liu et al. (2024b)
Amino acid derivatives				
Oral spermidine and spermine	Senescence-accelerated mice (brain)	↑ (p-AMPKα/t-AMPK)	↑ cognitive function	Xu et al. (2020)
Multiple indole derivatives	Cultured hippocampal cells and D-galactose-induced aging mice (brain)	↑ (p-AMPKα/t-AMPK)	↓ neuronal apoptosis, ↓ neurodegeneration	Yin et al. (2023)
Indole-3-Lactic Acid-treated cells	Senescent NCM460 intestinal epithelial cells	↑ (p-AMPKα/t-AMPK)	↓ oxidative stress and intestinal senescence	Fang et al. (2025)
Oral melatonin	Sleep fragmentation mouse model (white adipose tissue)	↑ (p-AMPKα/t-AMPK)	↓ metabolic dysfunction and inflammation	Hong et al. (2024)
Stress-induced elevated intestinal serotonin and serotonin-treated cells	Chronic restraint stress mouse model (colon) and cultured intestinal epithelial cells	↓ (p-AMPKα/t-AMPK)	↓ mitochondrial biogenesis and intestinal barrier function	Yang et al. (2024)
Trimethylamine-N-oxide-perfused heart	Mouse heart	↓ (p-AMPKα)	↓ contractile function	Naghipour et al. (2023)
Indoxyl sulfate-treated cells	Cultured neonatal rat cardiomyocytes	↓ (p-AMPKα/t-AMPK)	↑ oxidative stress and cardiomyocyte hypertrophy	Yang et al. (2015)
Subcutaneously-administered imidazole propionate and treated cells	8-wk-old mice, isolated bone marrow stromal cells	↓ (p-AMPKα/t-AMPK)	↑ adipocyte differentiation, ↓ osteoblast differentiation	Park et al. (2024)
Secondary bile acids				
Oral lithocholic acid	Mice (skeletal muscle), nematode worms, fruit flies	↑ (p-AMPKα/t-AMPK, p-ACC/t-ACC)	↑ lifespan, grip strength, running capacity, and muscle regeneration	Qu et al. (2024)

p-AMPKα/t-AMPK, the ratio of phosphorylated AMPK alpha (assessed via threonine 172) relative to total AMPK; p-ACC/t-ACC, the ratio of phosphorylated acetyl coenzyme A carboxylase (ACC) relative to total ACC. ↓, decreased; ↔, non-significant; ↑, increased.

#### 3.2.1 Short-chain fatty acids

Short-chain fatty acids (SCFAs) (e.g., acetate, butyrate, propionate) are some of the most extensively studied gut metabolites and are associated with numerous health benefits (Armet et al., 2022). Notably, short-chain fatty acids can also be directly obtained from food. For instance, fermented dairy products (e.g., cheese) contain several short-chain fatty acids, and the main component of vinegar is acetic acid (the acidic form of acetate). Many SCFAs (excluding acetate) are rapidly oxidized or used as gluconeogenic or lipogenic precursors in the liver (Morrison and Preston, 2016). Consequently, acetate is the primary SCFA capable of reaching systemic circulation and influencing broader host physiology (Morrison and Preston, 2016). Acetate has been shown to activate AMPK in the liver (Zhuge et al., 2025), skeletal muscle (Maruta et al., 2022), macrophages (Wada et al., 2025), and lung cells (Hu et al., 2024) in preclinical models, exemplifying that a single metabolite can have far-reaching effects (**Table 1**). Further, acetate inhibited atherosclerosis in mice, and these effects were reversed by an AMPK inhibitor (Compound C), demonstrating the possibility for AMPK to mediate the response (Wada et al., 2025). However, Compound C has known off-target effects. Hence, genetic approaches should be prioritized for validating AMPK-dependent effects.

#### 3.2.2 Polyphenol derivatives

Polyphenols are a class of phytochemicals characterized by multiple phenolic hydroxyl groups. They are broadly divided into two categories: flavonoids and non-flavonoids, each of which includes several subgroups. Many polyphenols have low bioavailability (as little as 5%) and must be converted into simpler forms to be absorbed and, therefore, exert their health effects (Zhang et al., 2021; Armet et al., 2022; Liu et al., 2024a). For example, isoflavone aglycones exhibit greater bioavailability because microbial cleavage of their glucose moieties increases lipophilicity and enhances absorption across the intestinal villi (Hsiao et al., 2020).

The gut microbiome is capable of metabolizing multiple classes of polyphenols, the unique structure of which (influenced by carbon chain length or other features) influences the ability of specialized enzymes to cleave the molecule (Kuziel et al., 2025). Ellagitannins, for instance, are converted into several urolithin subtypes in a manner that depends heavily on an individual’s gut microbiota composition (Shetty et al., 2024; Pidgeon et al., 2025). This microbiome-dependent pathway involves sequential ring-cleavage and dehydroxylation reactions and can vary greatly across individuals depending on their gut microbiota composition. Importantly, this type of microbial processing is shared across many dietary polyphenols, as gut bacteria often continue to degrade complex structures until only simple phenolics (those with a single benzene ring) remain. Other examples of compounds produced from microbial degradation include equol (from daidzein) (Soukup et al., 2021), dihydro-resveratrol (from resveratrol) (Bird et al., 2017), and a range of simple phenolics (caffeic, protocatechuic, quinic, and phenylacetic acids) from chlorogenic acid (Olthof et al., 2001; Raimondi et al., 2014).

Compounds with varying structural classifications appear to activate AMPK. Both p-coumaric acid (a hydroxycinnamic simple phenolic acid) and dihydro-resveratrol (a non-flavonoid polyphenol) activated hepatic AMPK in the liver of mice, alongside improvements in insulin sensitivity (Nguyen et al., 2021; Zhang et al., 2022; Lam et al., 2023). Similarly, equol (a flavonoid polyphenol) activated skeletal muscle AMPK in mice and improved serum glucose, cholesterol, and lipid levels (Cheong et al., 2014). Meanwhile, Yang et al. (2023) found that supplementing protocatechuic acid (a hydroxybenzoic simple phenolic acid) in the diet increased mouse skeletal muscle AMPK signaling, oxidative metabolism, and skeletal muscle fiber type conversion from fast-twitch type II fibers to slow-twitch type I fibers (Yang et al., 2023). Protocatechuic acid also activated gene expression indicative of mitochondrial biogenesis and fiber type conversion in cultured C2C12 myotubes, and this effect was completely abolished by Compound C (Yang et al., 2023), consistent with AMPK involvement.

#### 3.2.3 Amino acid derivatives

Gut microbiota also generate several important amino acid derivatives. For instance, the amino acid tryptophan (found in white meat, fish, dairy, and nuts) is a serotonin precursor. Short-chain fatty acids derived from the gut stimulate enterochromaffin cells to synthesize serotonin, which is subsequently converted into melatonin (Iesanu et al., 2022). The gastrointestinal tract is an important extra-pineal source of melatonin, and emerging evidence suggests bidirectional interactions between melatonin metabolism and the gut microbiota (Iesanu et al., 2022). Additionally, clinical trials indicate that exogenous melatonin supplementation may be beneficial for reducing inflammation (**Table 2**). In line with this, melatonin increased p-AMPKα/t-AMPK in mice white adipose tissue (Hong et al., 2024), while Compound C abrogated the protective effects of melatonin on oxidative-stress-induced apoptosis in bone mesenchymal stem cells (Fan et al., 2020).

**Table 2:** Systematic reviews and meta-analyses of human intervention or prospective cohort studies linking fermented foods and selected gut metabolites to health improvement.

Intervention	Participant characteristics	Study characteristics	Outcomes	References
Fermented Foods (e.g., cheese, fermented milk)	Healthy adults	Meta-analysis of 50 prospective cohort studies (> 3 million participants)	Lower risk of all-cause and cardiovascular disease mortality	Matalas et al. (2026)
Soy isoflavones (e.g., Equol)	Healthy adults > 18 years of age, mean age = 60	Meta-analysis of 16 RCTs (1386 participants)	Improved overall cognitive function and memory (SMD = 0.19 and 0.15; p ≤ 0.01)	Cui et al. (2019)
Equol	Women undergoing menopause	Meta-analysis of 5 RCTs (728 participants)	Lower hot flash scores (p = 0.01)	Daily et al. (2019)
Urolithin A	Healthy individuals	Systematic review yielding 5 RCTs (250 participants)	Increased strength and endurance, anti-inflammatory effect	Kuerec et al. (2024)
Melatonin	Individuals with and without chronic disease	Meta-analysis of 31 RCTs (1517 participants)	Anti-inflammatory effects (SMD = -1.11, -1.92, and -13.46 for IL-1, IL-6, and IL-8, respectively; p < 0.01 for all)	Cho et al. (2021)
Melatonin	Adults > 18 years of age	Meta-analysis of 12 RCTs (641 participants)	Reduced LDL cholesterol and triglyceride levels (SMD = 0.31 and 0.45; p = 0.049 and 0.006, respectively)	Loloei et al. (2019)
Acetic-acid-rich foods and beverages	Overweight or obese individuals or individuals with type II diabetes	Meta-analysis of 12 RCTs (622 participants)	Reduced triglyceride and fasting blood glucose levels (p ≤ 0.01)	Valdes et al. (2021)
Interventions with confirmed increases in SCFA levels	Healthy adults, obese/overweight adults, or adults with underlying conditions	Meta-analysis of 23 RCTs (1186 participants)	Reduced fasting insulin (SMD = -0.15; p = 0.04)	Pham et al. (2023)
Short-chain fatty acids	Individuals with obesity	Systematic review yielding 4 RCTs (83 participants)	3 of 4 studies reporting significant decreases in inflammatory cytokines	Eslick et al. (2022)

SMD, Standardized mean difference; RCT, Randomized controlled trial.

Other tryptophan metabolites include indoles and their derivatives, several of which are associated with health benefits (Ye et al., 2022) and are linked to AMPK activation (Yin et al., 2023; Fang et al., 2025). Spermidine, found primarily in the bloodstream as spermine, is another example of a protein derivative (from arginine) with the potential to protect against age-related increases in inflammation, metabolic dysfunction, and disease (Yu et al., 2024). Both spermine and spermidine were shown to improve cognitive function in senescence-accelerated mice while phosphorylating AMPK and inducing autophagy in brain tissue (Xu et al., 2020).

In contrast, several amino derivatives are reported to contribute to harmful health outcomes. The indole derivative, indoxyl sulfate, is often cited as nephro- and cardio-toxic (Yang et al., 2015; Ye et al., 2022). In line with this, indoxyl sulfate was shown to inhibit mouse cardiac AMPK signaling, leading to oxidative stress and cardiomyocyte hypertrophy (Yang et al., 2015). In addition, imidazole propionate was shown to drive atherosclerosis in mice, independent of altered lipid profiles (Mastrangelo et al., 2025). Following the pattern above, its health-antagonizing effects were associated with decreased AMPKα phosphorylation (Park et al., 2024).

#### 3.2.4 Other phytochemical derivatives

The gut microbiome can similarly enhance the bioavailability of even broader phytochemical classes. For example, sulforaphane is an isothiocyanate produced from the degradation of glucoraphanin by myrosinase enzymes upon tissue destruction (i.e., chewing) in raw cruciferous vegetables (Dmytriv et al., 2025). However, cooking destroys the enzyme and prevents sulforaphane production (Dmytriv et al., 2025). Alternatively, gut bacterial myrosinases can provide the enzymatic function and help produce bioactive levels of sulforaphane (Dmytriv et al., 2025). As for other phytochemicals, sulforaphane has been shown to improve a variety of health outcomes in preclinical models alongside AMPK modulation (Sun et al., 2020; Zhang et al., 2022).

#### 3.2.5 Secondary bile acids

Bile acids produced to aid fat absorption during the consumption of high-fat diets could be another modifying factor for disease risk. The gut microbiome metabolizes primary bile acids into secondary bile acids (Wu et al., 2021), which cumulatively represent the most abundant pool of gut metabolites (Wu et al., 2021). However, there are conflicting reports as to whether they aid or inhibit disease progression (Lajczak-McGinley et al., 2020; Qu et al., 2024; Chen et al., 2025).

Lithocholic acid (LCA) is a secondary bile acid shown to increase in response to caloric restriction in mice (Qu et al., 2024). Importantly, this effect was not seen in germ-free or antibiotic-treated mice, indicating the microbiome’s role in its production (Qu et al., 2024). LCA was subsequently shown to activate AMPK or its analogs and extend lifespan and healthspan in mice, *C. elegans*, and *D. melanogaster* in an AMPK-dependent manner (Qu et al., 2024). In particular, LCA improved running distance, duration, and grip strength in aged mice (Qu et al., 2024). AMPK activation was confirmed via downstream ACC signaling as well as reduced mTORC1 (mammalian target of rapamycin) signaling, which may partially explain the healthspan-promoting benefits. Further, AMPK-dependency was confirmed via muscle-specific AMPK knockout in mice (Qu et al., 2024).

### 3.3 Bacterial endotoxins

Intriguingly, even lipopolysaccharide (LPS), a bacterial endotoxin released from the death of gram-negative bacteria, may affect AMPK and mediate gut microbiome effects on health (Cani et al., 2007). Disruption of the intestinal barrier allows LPS to enter the bloodstream. LPS has been shown to dose-dependently inhibit AMPK through Thr172 dephosphorylation in mouse lung tissue (Fan et al., 2018). This process is likely mediated by toll-like receptors (TLRs), since LPS engagement of TLR4 diminishes AMPK activation capacity (Tadie et al., 2012). Further, since AMPK can negatively regulate NF-kß (Chen et al., 2018), this inhibition could be at least partially responsible for the pro-inflammatory nature of LPS.

Conversely, AMPK inhibits NF-kβ (Chen et al., 2018), providing an anti-inflammatory benefit. For example, a multi-strain probiotic modestly (but significantly) increased p-AMPK/t-AMPK, inhibited NF-kβ and pro-inflammatory cytokine expression, and promoted tight junction formation in Caco-2 cells (Han et al., 2023). It is unclear exactly what components mediate these effects, but it may be due to cell wall components, as suggested by others (Shi et al., 2022).

## 4 Effects of AMPK activation on the gut microbiome

Oftentimes, gut-tissue axes are thought of as a bidirectional communication system. Interestingly, AMPK activity and the microbiome may exhibit a similar reciprocal relationship. Metformin-induced AMPK activation in high-fat, high-sucrose-fed mice reduced colonic levels of secondary bile acids (Bravard et al., 2021). This was associated with altered gut microbial composition, including an increase in *Akkermansia muciniphila* (Bravard et al., 2021). Others similarly show that metformin can alter gut microbiota (Xu et al., 2025). However, these results may also be due to direct interactions between metformin and gut microorganisms. The specific effect of AMPK versus microbiota-mediated relationships is corroborated by intestinal tissue-specific AMPKα1 knockout in mice, which resulted in significant shifts in gut microbial composition, an increase in some bacterial metabolites (e.g., methylglyoxal), and a reduced production of antimicrobial peptides (Zhang et al., 2022). Additionally, fecal microbiota transplantation from the knockout mice resulted in increased fecal and serum levels of methylglyoxal in recipient mice, confirming the importance of the microbiota for altering the metabolome in response to intestinal AMPKα1 knockout (Zhang et al., 2022).

## 5 Mechanisms of altered AMPK activity

**Figure 2:**
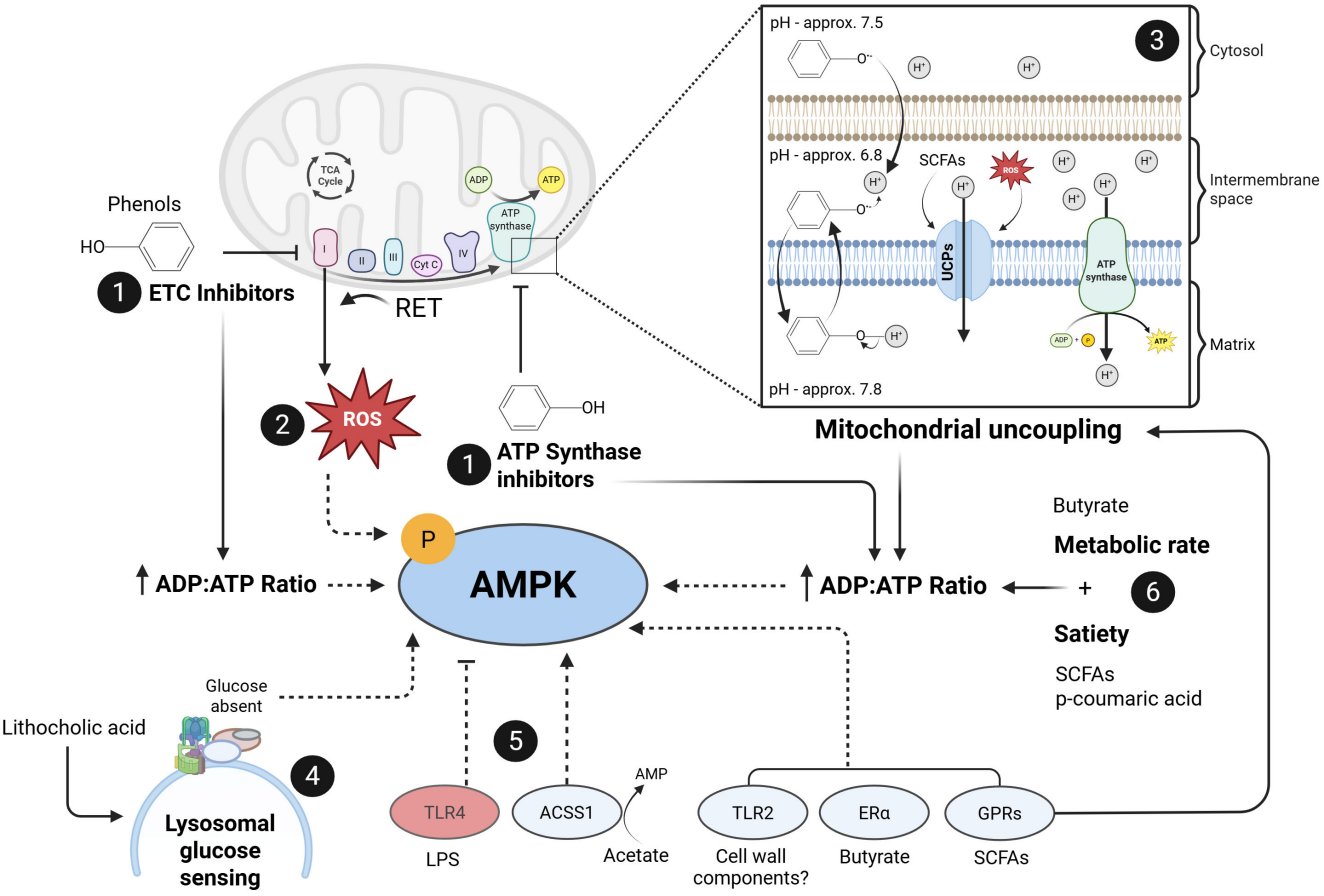
Mechanisms by which gut metabolites may influence AMPK activity. 1) Inhibiting mitochondrial function can increase AMP or ADP levels and activate AMPK. 2) ROS can directly activate AMPK. 3) Uncoupling proteins and protonophores can lead to mitochondrial uncoupling, increased AMP or ADP levels, and AMPK activation. 4) Absence of glucose can activate AMPK independent of altered AMP or ADP levels. 5) Several signaling pathways can indirectly or directly activate or inhibit AMPK. 6) Increased satiety coupled with upregulated metabolic rate can result in net negative energy balance and AMPK activation. AMPK, adenosine monophosphate-activated protein kinase; ETC, electron transport chain; RET, reverse electron transport; ROS, reactive oxygen species; UCPs, uncoupling proteins; SCFAs, short-chain fatty acids; TLR4, toll-like receptor 4; ACSS1, acyl-CoA synthetase short-chain family member 1; ERα, estrogen receptor alpha; GPRs, G-protein coupled receptors. Created in Biorender.com.

The effects of the gut microbiome on AMPK signaling may be mediated by several direct or indirect pathways, including the following: altered mitochondrial bioenergetics, interactions with other signaling pathways, altered energy balance, and phosphorylation of AMPK at inhibitory sites (**Figure 2**).

### 5.1 Altered mitochondrial bioenergetics

The canonical pathway for AMPK activation involves the sensing of low-energy states. As such, mitochondrial bioenergetics are closely intertwined with AMPK activity. One pathway through which AMPK becomes activated involves a mild increase in mitochondrial uncoupling. Under normal conditions, mitochondria generate energy by establishing a proton gradient across the inner membrane, which then drives ATP synthase to convert ADP and inorganic phosphate into ATP. In contrast, mitochondrial uncouplers can allow protons to bypass ATP synthase, releasing heat instead of generating ATP (Klaus et al., 2011). This leads to an increase in AMP: ATP or ADP: ATP ratios and can activate AMPK (Klaus et al., 2011). Multiple short-chain fatty acids have been shown to increase the expression of uncoupling proteins (UCP1, UCP2, and UCP3), thereby activating AMPK (He et al., 2020). Conversely, indoxyl sulfate was shown to downregulate UCP2, resulting in AMPK inhibition in cultured rat cardiomyocytes (Yang et al., 2015). Further, AMPK inhibition helped to maintain UCP2 downregulation (Yang et al., 2015).

Protonophores provide another means for mitochondrial uncoupling. Protonophores have a unique structure that allows them to transfer protons across the mitochondrial membrane. Many phenolic compounds are especially effective protonophores because of their lipophilicity and the presence of resonance structures that yield stable molecules when deprotonated (Sandoval-Acuña et al., 2014; Stevens et al., 2018). In the slightly more basic conditions of the cytosol (pH ≈ 7.5), these molecules deprotonate (Stevens et al., 2018). They are subsequently passed into the mitochondrial inner membrane, where they are then re-protonated in the more acidic environment (pH ≈ 6.8) (Stevens et al., 2018). The acidic version of the compound can then pass into the mitochondrial matrix, releasing a hydrogen ion and returning to the inner membrane. This process can repeat and continue to transfer hydrogen ions across the inner membrane, thereby bypassing ATP synthase and reducing ATP production (Stevens et al., 2018). Phenolic compounds could also influence AMPK activity by inhibiting mitochondrial complexes to decrease ATP production (Sandoval-Acuña et al., 2014). Conversely, TMAO increased mitochondrial complex I and II and maximal respiratory fluxes (Naghipour et al., 2023). The increase in energy production was associated with a reduced expression of phosphorylated AMPK.

Another pathway for AMPK activation is by mildly increasing mitochondrial reactive oxygen species (mROS) production. Spermidine increased mROS and activated AMPK in mouse bone marrow-derived macrophages (Liu et al., 2020). One proposed mechanism for ROS-induced AMPK activation is a decline of mitochondrial energy production to increase the AMP: ATP ratio and thereby activate AMPK through its canonical energy-sensing pathway (Hinchy et al., 2018). This can be explained by the effect of ROS (superoxide) on increasing UCP expression and, therefore, increasing mitochondrial uncoupling (Echtay et al., 2002). Superoxide could also directly act as a protonophore (Brookes, 2005). However, experimental evidence is still lacking. Alternatively, others show that ROS can affect AMPK activity independent of changes to the AMP: ATP ratio (Emerling et al., 2009; Rabinovitch et al., 2017).

### 5.2 Interactions with other signaling pathways

Further, gut metabolites can influence AMPK indirectly through interactions with several signaling pathways. For instance, acetate is converted into AMP and acetyl-coA by acyl-CoA synthetase short-chain family members 1 and 2 (ACSS 1 and 2) in the mitochondria (Zhuge et al., 2025). *Akkermansia-muciniphila* is suggested to upregulate ACSS1 to promote AMP synthesis and activate AMPK (Zhuge et al., 2025). However, the exact mechanism remains unclear.

Many metabolites also interact with G-protein coupled receptors (GPRs) to activate AMPK. GPR43 is a well-known SCFA receptor that can induce AMPK activation (Yoshida et al., 2019; Hu et al., 2024), while indole derivatives have been reported to phosphorylate AMPK via GPR30 (Yin et al., 2023). The effect of GPRs may be at least partially mediated by increased UCP expression (Hu et al., 2016).

Alternatively, estrogen receptors interact with AMPK and may mediate the effects of SCFAs on AMPK activity. Butyrate interacts with estrogen receptor alpha (ERα) to increase skeletal muscle AMPKα phosphorylation and reduce metabolic dysfunction in a mouse model of diet-induced obesity and menopause (Fu et al., 2023). This effect was dampened by inhibiting ERα (Fu et al., 2023), indicating that the binding of butyrate to ERα likely mediates its effects on AMPK signaling and metabolic outcomes. The data also point to the potential sex-specific impacts of gut metabolites and provide opportunities for precision nutrition.

In another example, Toll-like receptors (TLRs) inherently respond to microbes as part of the innate immune defense and can modulate AMPK. Pasteurized *Akkermansia muciniphila* (a probiotic with potential health benefits (Zhou, 2017)) increases p-AMPKα/t-AMPK via a TLR2-dependent mechanism (Shi et al., 2022). The authors propose that the interaction between cell wall components and TLR2 is responsible for this result (Shi et al., 2022).

Lastly, Qu et al. (2024) report that lithocholic acid activates AMPK via the lysosomal glucose-sensing pathway. This pathway senses glucose levels through glycolytic intermediates and can function independently of altered AMP levels (Lin and Hardie, 2018). These pathways are likely not exclusive, and gut metabolites may influence many other pathways that affect AMPK activity.

### 5.3 Phosphorylation of AMPK at inhibitory sites

AMPK phosphorylation at its Thr 172 residue (AMPKα) is reported most often, since it is a primary mechanism for covalent AMPK activation (Hardie et al., 2016; Willows et al., 2017; Steinberg and Hardie, 2023). However, several other phosphorylation sites can influence the activation capacity of AMPK by other kinases and the effectiveness of the aforementioned strategies (Hawley et al., 2014; Steinberg and Hardie, 2023). One exemplary study found that imidazole propionate impairs metformin-induced AMPKα phosphorylation in mice by p38γ/AKT-induced phosphorylation of inhibitory serine 485/495 residues on AMPKα1/α2 (Koh et al., 2020). Given that insulin stimulates AKT phosphorylation, the effects of gut metabolites on overall cardiometabolic health (via AMPK-dependent and AMPK-independent pathways) could impact future AMPK activation capacity. This is in line with evidence demonstrating that individuals with obesity or metabolic syndrome have reduced AMPKα phosphorylation in skeletal muscle following resistance exercise relative to healthy controls (Sriwijitkamol et al., 2007; Lee-Young et al., 2010; Layne et al., 2011). These studies suggest that modulating the gut microbiome may enhance the effectiveness of AMPK-activating interventions (e.g., exercise) in individuals with reduced AMPK sensitivity, such as older adults (Salminen et al., 2016), trained athletes (Nielsen et al., 2003), or those with metabolic syndrome and/or obesity (Sriwijitkamol et al., 2007; Layne et al., 2011).

### 5.4 The gut-brain axis and energy balance

The gut microbiome’s effect on energy balance may also indirectly shape AMPK activity (Singh and Chelikani, 2019). In particular, there is a close relationship between the endocrine control of food intake and AMPK, as reviewed in detail elsewhere (Stark et al., 2013; Huynh et al., 2016; Steinberg and Hardie, 2023). Briefly, several factors, including glucagon-like peptide-1 (GLP-1) receptor agonists and leptin, can inhibit hypothalamic AMPK, which is central to their effects on reducing food intake (Huynh et al., 2016). Meanwhile, these factors are reported to *increase* AMPK phosphorylation in brown adipose tissue, driving thermogenesis (Huynh et al., 2016). Additionally, vagal afferent signaling complements neuroendocrine processes and is a key link between intestinal and central effects. In particular, short-chain fatty acids may reduce food intake through vagal afferent signaling (Goswami et al., 2018).

As for endocrine signals, gut metabolites may exert differential effects on hypothalamic and peripheral AMPK activity, reinforcing a negative energy balance. P-coumaric acid, for example, inhibits hypothalamic AMPK signaling, while also increasing peripheral AMPK signaling in mice (Nguyen et al., 2021). This result was associated with enhanced leptin signaling and satiety, reduced food intake, and improved glucose homeostasis (Nguyen et al., 2021).

A similar pattern is observed with acetate. Stable isotope tracers confirmed that colonic acetate crosses the blood-brain barrier and inhibits hypothalamic AMPK activity in mice while increasing the expression of satiating hormones (GLP-1 and peptide YY) and decreasing food intake (Frost et al., 2014). Others demonstrate that acetate also enhances peripheral AMPK signaling (Maruta and Yamashita, 2020; Hu et al., 2024; Zhuge et al., 2025). This dual action of gut metabolites on energy expenditure and satiety could promote further AMPK activity in peripheral tissues by maintaining a lower energy balance. However, this remains a hypothesis and needs further confirmation in human studies.

## 6 Translational potential

Many of the interventions discussed above and their related metabolites have also been shown to have beneficial health effects in human clinical trials. For instance, probiotic-rich cheese and yogurt have been shown to phosphorylate AMPK in preclinical models (Kim et al., 2023; Sun et al., 2024), while fermented dairy is accordingly associated with reduced all-cause and cardiovascular disease mortality (Matalas et al., 2026). Multiple lines of preclinical evidence similarly indicate that SCFAs, such as acetate, phosphorylate AMPK across diverse tissues, which is consistent with improved metabolic health in multiple systematic reviews and meta-analyses of randomized controlled trials (**Tables 1** and **2**). Collectively, these and other examples are compatible with the proposed gut-AMPK axis and highlight the potential translational value of the concepts discussed herein. However, they do not yet prove a gut-AMPK axis in humans.

## 7 Limitations and safety considerations

Although promising, targeting AMPK currently comes with inherent practical challenges. As in many preclinical studies, Thr172 phosphorylation is typically used as a proxy for AMPK activity in human clinical trials (Nielsen et al., 2003; Boyle et al., 2011; Timmers et al., 2011; Wan et al., 2014; Fritzen et al., 2015; Fuller et al., 2017; Godara et al., 2020). However, questions remain surrounding whether Thr172 phosphorylation in biopsied tissue or isolated immune cells reflects *in vivo* AMPK activity. Moreover, AMPK activity cannot currently be measured directly in humans *in vivo*, and assessing it through tissue biopsies involves invasive procedures. Although changes (pre-post 26 wk dietary intervention) in biopsied adipose tissue AMPK Thr172 phosphorylation have previously been correlated with changes in the HOMA-IR measure of insulin resistance in humans (Fritzen et al., 2015), the development of standardized, fully quantitative measures of *in vivo* AMPK activity would enhance the validity of such studies (Dassoff, 2026).

Targeting AMPK also carries certain risks. AMPK is a central node, with multiple inputs and outputs. This low specificity offers broad-spectrum therapeutic potential, but it also increases the risk of off-target effects, such as pathological cardiac hypertrophy (McGee and Hargreaves, 2020). This concern parallels those associated with pharmacological mitochondrial uncouplers (e.g., 2,4-dinitrophenol), which carry the risk of non-mitochondrial effects and systemic toxicity (Goedeke and Shulman, 2021).

The rhythmicity and duration of AMPK activation are, similarly, potential concerns. Such concerns are highlighted in a recent review (Dassoff, 2026). One study of the metabolite phenylacetylglutamine (PAGln)—downstream of the gut-derived phenylacetate—illustrates this complexity (Yang et al., 2025). PAGln has been reported to promote DNA damage by phosphorylating the mitochondrial fission protein dynamin-related protein 1 (Drp1), resulting in increased mitochondrial reactive oxygen species and DNA damage (Yang et al., 2025). In contrast, others report that moderate Drp-1 phosphorylation may extend healthy lifespan in model organisms (Rana et al., 2017). These findings imply that AMPK activation may be beneficial, provided it is moderate and not accompanied by DNA damage (Dassoff, 2026). This nuance is important, given that AMPK activation is also an adaptive response to genotoxic stress, due to its role in DNA damage repair (Wu et al., 2013; Szewczuk et al., 2020; Steinberg and Hardie, 2023).

From a practical standpoint, jointly assessing AMPK activation and DNA damage would provide a more informative and complementary endpoint (Dassoff, 2026). Meanwhile, mild uncoupling and/or sporadic AMPK activation (akin to diet and exercise) are potential strategies to reduce risks while retaining their broad therapeutic benefits (McGee and Hargreaves, 2020; Goedeke and Shulman, 2021). Given the challenges in precisely defining what levels or durations of AMPK activation are adaptive or maladaptive, interventions should currently be limited to levels routinely encountered throughout a typical lifestyle.

## 8 Future research

Future research should focus on establishing the Gut-AMPK relationship and its physiological significance in humans. Conclusive evidence requires multiple human clinical trials demonstrating the effects of differing microbiota-targeted interventions on AMPK activity across cells or tissues (e.g., immune, adipose, muscle). AMPK activity should be determined through both AMPK phosphorylation, with specific mention of the subunits involved, alongside additional downstream validation or measures of direct AMPK kinase activity. Meanwhile, conclusively establishing a bidirectional axis will require further research into whether AMPK-activating interventions, such as moderate-to-vigorous-intensity exercise or metformin, consistently influence the gut microbiome. Importantly, trials should include mechanistic and phenotypic measures within the same study design to better establish a microbiome-AMPK-phenotype causal chain in humans.

Establishing a gut-AMPK axis could be beneficial in several ways. Although questions remain regarding optimal AMPK-activation strategies, AMPK may play an important role in modulating processes that reduce disease risk. Future trials may consider exploring interactions between the microbiome and AMPK as a mechanistic explanation for dietary interventions with established health benefits (e.g., the Mediterranean diet).

Eventually, AMPK might be considered a useful target for more novel microbiota-targeted interventions. AMPK could be an especially valuable preclinical target for rapidly screening a wide range of potentially beneficial compounds. For example, the effects of metabolites on AMPK or related pathways can be screened via computational structure-based approaches (Chen et al., 2024; Jeon et al., 2025). These workflows can subsequently be integrated with *in vitro* cell culture approaches to screen for the effects of metabolites on cellular responses (i.e., AMPK activity) (Zhang et al., 2025) and ultimately translated into human clinical trials to confirm clinical benefits.

Possible interventions may include metabolites themselves or combinations of pre- and probiotics (synbiotics). AMPK-activating metabolites could equally be derived indirectly from the diet by strategically manipulating the microbiome to increase its capacity to produce these compounds. This approach has the advantage of inherently producing a wider range of bioavailable and bioactive compounds in physiological doses while simultaneously promoting gut and systemic health (Kibe et al., 2014). This suggestion aligns closely with that of others proposing the development of microbiota-directed foods (Barratt et al., 2017). Importantly, the development of such foods will depend on many factors, including inter-individual variability and microbiome heterogeneity.

## 9 Concluding remarks

The gut microbiome has long been an enticing direction for nutrition research because of its significant effect on diverse facets of health. However, the vastness of the field has posed challenges for outlining mechanistic pathways and efficiently generating actionable insights. Evidence presented throughout this review highlights that gut metabolites may broadly influence host AMPK activity across skeletal muscle, liver, bone marrow, brain, heart, intestine, lung, immune, and adipose cells or tissues. Moreover, many of the health benefits of gut-derived molecules may depend on AMPK activation. Taken together, this body of evidence supports that AMPK is a plausible signaling node with the potential to mediate the effects of the gut microbiome on downstream health outcomes. However, much of the existing evidence is preclinical, and clinical trials are an increasing priority to confirm this mechanistic hypothesis. Ultimately, these insights may help to advance nutrition far beyond its trial-and-error roots, help resolve existing nutrition controversies, and inform the design of novel functional foods. Advancing our knowledge of the interactions between these factors, the gut microbiome, and AMPK activity holds great potential to prevent disease and enhance healthspan.
